# Kikuchi-Fujimoto Disease Complicated by Rheumatoid Arthritis, Type 1 Diabetes Mellitus, and Hypothyroidism

**DOI:** 10.7759/cureus.21008

**Published:** 2022-01-07

**Authors:** Soroush Shahrokh, Ammar Hasan, Salman Alim, Michelle Hebert, Khulood Rizvi

**Affiliations:** 1 Internal Medicine, HCA Houston Healthcare Kingwood/University of Houston College of Medicine, Kingwood, USA; 2 Critical Care Medicine, HCA Houston Healthcare Kingwood/University of Houston College of Medicine, Kingwood, USA; 3 Pathology, HCA Houston Healthcare Kingwood/University of Houston College of Medicine, Kingwood, USA; 4 Infectious Disease, HCA Houston Healthcare Kingwood/University of Houston College of Medicine, Kingwood, USA

**Keywords:** autoimmune hypothyroidism, neutropenic fever, kikuchi-fujimoto disease, lymphadenopathy, rheumatoid arthritis, lymphoma, lymphoproliferative disorders

## Abstract

Kikuchi-Fujimoto disease (KFD) is a rare, benign, self-limited syndrome characterized by tender lymphadenopathy and low-grade fever. It may also present with rash, arthritis, fatigue, and splenomegaly. Data on the disease is limited, and its etiology remains largely unknown.

Here, we present the case of a 30-year-old female with a medical history of rheumatoid arthritis (RA), previously treated with etanercept, type 1 diabetes mellitus (DM-1), and Hashimoto’s hypothyroidism; she was brought in to an emergency department (ED) in Houston after a generalized tonic-clonic seizure and loss of consciousness. She was hypoglycemic, which was thought to have caused her DM-1 and seizure. CT scan of her chest showed multiple enlarged lymph nodes throughout the neck, superior mediastinum, and axilla, along with interstitial edema and bilateral pleural effusions. She was treated with dextrose drip and regained her consciousness. However, she had persistent pancytopenia, low-grade fever, and tender axillary lymphadenopathy. Infectious workup for tuberculosis (TB), human immunodeficiency virus (HIV), herpes simplex virus (HSV), Epstein-Barr virus (EBV), and parvovirus B-19 were negative. Her bone marrow biopsy revealed iron-deficiency anemia, while excisional axillary lymph node biopsy showed extensive necrosis consistent with KFD. She was treated with supportive care. Her neutrophilic fever resolved, and she was discharged home after 48-hours of remaining afebrile. Six months after her hospitalization, the patient remained well, and her complete blood count showed no abnormalities.

Due to the non-specific clinical features and laboratory findings of KFD, it is commonly misdiagnosed as infectious, autoimmune, or malignant lymphadenitis, leading to excessive diagnostic tests and unnecessary treatments. Physicians need to be cognizant of KFD and consider it in young patients presenting with tender lymphadenopathy, low-grade fevers, and leukopenia. To our best knowledge, this is the first reported case of a patient with concurrent RA, Hashimoto’s hypothyroidism, and KFD. This report elucidates the autoimmune nature of KFD and its association with other autoimmune diseases.

## Introduction

Kikuchi-Fujimoto disease (KFD), also known as Kikuchi histiocytic necrotizing lymphadenitis, is a rare and poorly described disease characterized by tender lymphadenopathy and low-grade fever [1]. KFD was first described in 1972, with only a limited number of cases reported [2-3]. Although KFD was initially thought to predominantly affect women with a male-to-female ratio of 1:4, recent studies have shown a significantly more equal gender distribution with a male-to-female ratio of 1: 1.26 [4-5]. The etiology of KFD remains largely unknown, with loose and poorly described association with previous viral infections and autoimmune diseases [1,6]. Here, we describe a rare case of a 30-year-old female with a history of rheumatoid arthritis (RA), Hashimoto’s thyroiditis, and type 1 diabetes mellitus (DM-1) who presented with seizures and was found to have mediastinal and axillary lymphadenopathy, low-grade fever, and pancytopenia, which was diagnosed as KFD.

## Case presentation

A 30-year-old female with a history of RA, DM-1 on insulin, and Hashimoto’s hypothyroidism was brought to an emergency department (ED) in Houston after a generalized tonic-clonic seizure and loss of consciousness. According to her family, the patient felt fatigued several days before her seizure and complained of painful right axillary lymphadenopathy and low-grade fever, and night sweats for two weeks. She had no pets or recent travel history. The patient was diagnosed with RA three years ago. She was treated with weekly etanercept injections for one year, with her disease in remission until three months prior to her hospitalization when she lost her insurance coverage.

Upon arrival, the patient was febrile with a temperature of 104.0 F, tachycardia, and tachypnea. On physical exam, she was lethargic and disoriented, had oral thrush, prominent right axillary lymphadenopathy, and mild hepatomegaly but no splenomegaly. Her laboratory results showed hypoglycemia, elevated creatinine kinase (CK), lactic acidosis (LA), leukopenia, and microcytic anemia (Table 1). CT scans of the head, chest, abdomen, and pelvis showed multiple enlarged lymph nodes in the neck, superior mediastinum, and axilla. CT-angiogram of her chest showed moderate interstitial edema, bilateral pleural effusions, small pericardial effusion, peri-aortic lymphadenopathy, and mild hepatomegaly.

**Table 1:** Patient’s laboratory results on hospital admission, day 6 of her hospital stay, and discharge. Hb: hemoglobin; Plt: platelet count; MCV: mean corpuscular volume; WBC: white blood cells; ANC: absolute neutrophil count; TIBC: total iron-binding capacity; CK: creatinine kinase; LA: lactic acidosis

Laboratory Study	Day 1	Day 6	Discharge	Reference Range
Hb	6.3 x 10^3/mm3	12.3 x 10^3/mm3	12.3 x 10^3/mm3	12.0-15.5x 10^3/mm3
Plt	354 x 10^3/mm3	425 x 10^3/mm3	425 x 10^3/mm3	150-350 x 10^3/mm3
MCV	66 fl	70 fl	74 fl	80–100 fl
WBC	3.2 x 10^3/mm3	1.0 x 10^3/mm3	4.0 x 10^3/mm3	4.5-11.0 x 10^3/mm3
ANC		0.60 x 10^3/mm3		2.5-8.0 x 10^3/mm3
Iron	63 mcg/dL			60-170 mcg/dL
TIBC	163 mcg/dL			250-400 mcg/dL
Ferritin	594 ng/mL			12-300 ng/mL
Glucose	52 mg/dL	170 mg/dL	130 mg/dL	80-110 mg/dL
CK	461 U/L	161 U/L		22-198 U/L
LA	2.4 mmol/L	1.0 mmol/L	0.8 mmol/L	0.5-1.0 mmol/L

The patient was admitted to the intensive care unit (ICU) for intravenous (IV) dextrose drip and neurological monitoring. Additional labs obtained in the ICU showed a normal serum iron, low total iron-binding capacity (TIBC), high ferritin with normal folate and cobalamin levels.

She was diagnosed with anemia of chronic disease and received two units of packed red blood cells and IV-iron transfusions. She was treated with piperacillin/tazobactam. Neurology was consulted and MRI of the brain and electroencephalogram (EEG) were obtained, both of which were normal. Her seizures and altered mental status were attributed to metabolic encephalopathy because of hypoglycemia, and after the improvement of her mentation, she was transferred to the intermediate care unit (IMU). The patient’s repeat labs showed normal lactic acid and improved hemoglobin. However, she continued to have tender lymphadenopathy, with her leukocyte count continuing to downtrend six days after hospital admission.

Despite receiving broad-spectrum antibiotics, the patient remained febrile with worsening neutropenia. Because of concern for malignancy, both infectious disease and oncology services were consulted, and an initial diagnosis of lymphoma versus viral lymphadenitis versus RA-induced lymphadenopathy was considered. The patient was placed in isolation and was started on valacyclovir and fluconazole for her oral thrush. Her serum inflammatory markers and auto-antibodies including C-reactive protein, antinuclear antibody (ANA), anti-double-stranded-DNA (anti-DS-DNA) antibody, and anti-Smith antibody levels were all within normal range. Infectious workup for tuberculosis (TB) with interferon-gamma release assay, human immunodeficiency virus (HIV) antigen/antibody 4th generation assay, herpes simplex virus (HSV) 1 & 2 serology, cytomegalovirus (CMV) serum polymerase chain reaction (PCR), hepatitis virus serum serology, Epstein-Barr virus (EBV), and parvovirus B-19 serum serology were all negative. A peripheral blood smear (PBS), thoracentesis, bone marrow biopsy, and excisional lymph node biopsy from the right axilla were obtained and sent for pathologic evaluation.

**Figure 1:**
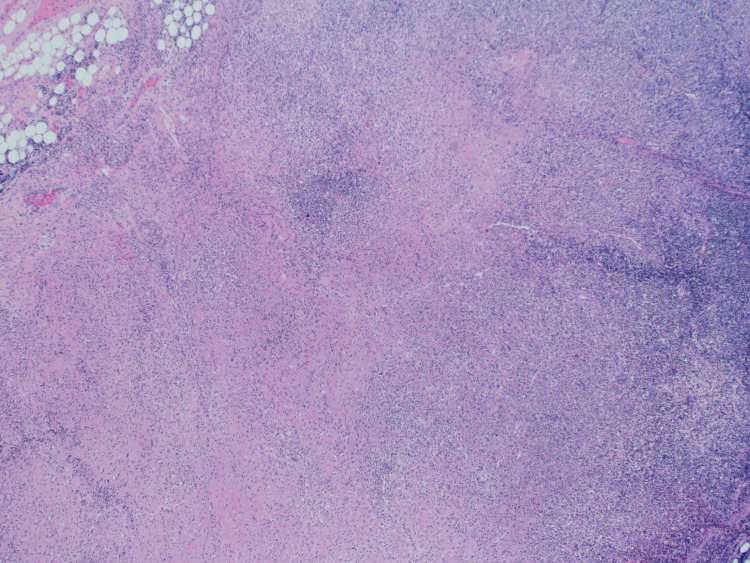
Bone-marrow biopsy showing a normocellular bone marrow with trilineage hematopoiesis, mildly left shifted granulopoiesis and polyclonal plasmacytosis, along with decreased iron stores. There is no evidence of increased blast cells, significant dysplasia or lymphoproliferative disorders observed.

**Figure 2:**
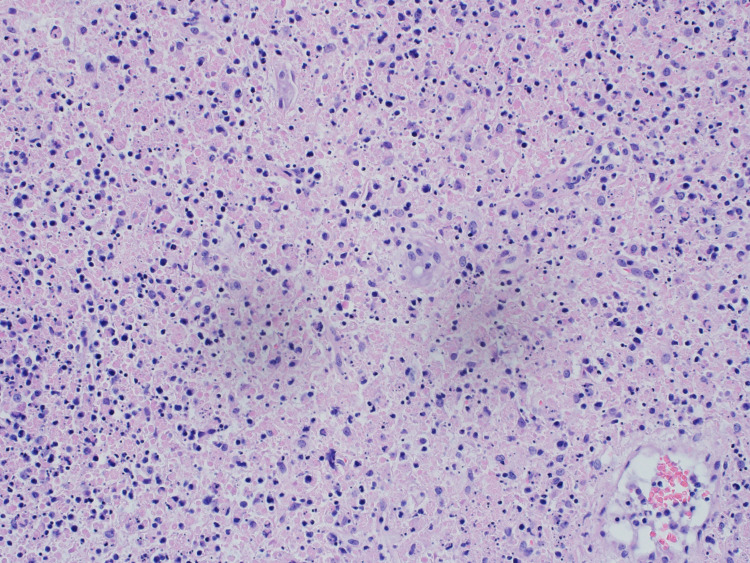
Patient’s right axillary lymph node biopsy hematoxylin and eosin (H&E) stain showing the presence of necrosis with nuclear debris, apoptotic bodies, and scattered clusters of foamy macrophages with palisading macrophages at the edge of the foci of necrosis. There is no evidence of lymphoma or an infectious (viral, fungal or bacterial) process identified, with a final diagnosis of Kikuchi-Fujimoto lymphadenopathy and an alternative differential diagnosis of systemic lupus erythematosus.

The patient’s PBS showed microcytic, hypochromic anemia with lymphopenia and absolute neutropenia. Bone marrow biopsy showed a normocellular bone marrow with decreased iron stores but no evidence of increased blast cells, dysplasia, or lymphoproliferation (Figure 1). Flow cytometry analysis of the bone marrow aspiration showed no immunophenotypic abnormalities. Microscopic examination of the lymph-node biopsy revealed extensive necrosis and scattered clusters of palisading macrophages (Figure 2). No evidence of lymphoma or an infectious process was identified, and the results were consistent with KFD.

The patient was diagnosed with KFD lymphadenopathy. She received two doses of filgrastim, after which her leukocyte counts improved. She was placed on an insulin regimen and oral iron tablets. Her fever resolved, and her lymphadenopathy subsided, and she was discharged home after 48-hours of remaining afebrile. In a six-month follow-up, the patient reported no complaints since her discharge from the hospital and was found to have normal physical exam and lab findings.

## Discussion

KFD is a benign and self-limited disease characterized by subacute onset of tender lymphadenopathy and low-grade fevers [1]. It was initially described in 1972 simultaneously by Masahiro Kikuchi and Fujimoto et al. in two Japanese women with cervical lymphadenopathy, low-grade fever, rash, and arthralgias [2-3]. KFD also commonly causes diffuse rash, arthritis, fatigue, and hepatosplenomegaly, while it less commonly causes thyroiditis, encephalitis, seizures, pleuritis, pericarditis, hepatitis, and anti-phospholipid syndrome leading to multi-organ failure [7,8,9-12]. Laboratory and serologic studies are often normal but may show leukopenia (in 43% of patients), thrombocytopenia, anemia, or atypical lymphocytosis (in 25% of patients). Patients may also have a slightly elevated erythrocyte sedimentation rate (ESR) and liver function tests (LFTs). However, auto-antibodies, i.e., rheumatoid factor (RF), ANA, and anti-DS-DNA antibodies, are always within the normal range [12]. Bone marrow biopsy often shows macrophagocytosis without atypia, while lymph node biopsy shows necrosis and histiocytosis [12-13]. Because of its benign and self-limited nature, KFD is managed by supportive care and observation [14]. However, short tapers of steroids and hydroxychloroquine may be used in severe cases, although their efficacy is only based on anecdotal evidence [14].

The etiology of KFD is not well elucidated [1,6]. Previously, it was postulated that KFD has a viral etiology [13]. This theory was supported by the similar histopathologic features seen in viral lymphadenitis and KFD, including paracortical necrosis and T-cell predominance in the affected lymph nodes, reactive lymphocytosis on PBS, and macrophagocytosis in bone marrow biopsy [6,12]. The most common viral infections associated with KFD are HSV, HIV, EBV, parvovirus B19, paramyxovirus, and CMV [13]. However, more recent studies have disputed this association, as no viral organisms were found in patients with KFD [14].

Other studies have suggested an autoimmune cause for KFD [15-17]. This theory has been more widely accepted than the previously proposed viral etiology since there has been a close link between clinical manifestations and histopathologic features of KFD and autoimmune disorders, i.e., systemic lupus erythematosus (SLE), Castleman disease, Still’s disease, and Sjogren’s syndrome [16-19]. Notably, a close relationship is observed between KFD and SLE, leading some to propose KFD as a self-limited case of SLE [17]. Other studies have postulated that KFD may be an overactive T-cell-mediated immune response to different antigens in genetically susceptible patients. This theory is supported by the prevalence of human leukocyte antigen (HLA) class II alleles, specifically HLA-DPA1 and HLA-DPB1, in both KFD and SLE patients [15]. Dumas et al. reported that amongst 91 KFD patients he studied, 11 (12%) would eventually develop SLE. These cases likely represent lupus lymphadenitis, as the two disorders are histologically indistinguishable in some cases [16]. However, unlike SLE, KFD is not associated with the presence of ANA, anti-Smith, or anti-DS-DNA antibodies [12].

Given our patient’s history of RA and recent immunosuppressive therapy with etanercept, hematologic malignancies and especially lymphoma were high on our differential diagnoses. This was further supported by our patient’s clinical presentation (constitutional symptoms such as fever, night sweats, and weight loss), physical exam findings (i.e., mediastinal and axillary lymphadenopathy), and serologic abnormalities (i.e., pancytopenia), all of which are characteristics of lymphomas. However, the timeline of her symptoms and the painful nature of her lymphadenopathy were incompatible with lymphoma. While reactive (i.e., infectious or autoimmune) lymphadenopathies are characterized by severe pain and tenderness, malignant lymphadenopathies are typically painless. Additionally, malignant lymphadenopathies often have an insidious onset and develop gradually over several months; reactive lymphadenopathies, on the other hand, typically develop either acutely or sub-acutely, within several days to weeks. These factors led us to look for other etiologies as the cause of our patient’s symptoms.

Furthermore, although we initially attributed our patient’s neurologic symptoms to metabolic encephalopathy caused by hypoglycemia, KFD may have been a major culprit. However, multiple studies have reported neurologic involvement by KFD, with aseptic meningoencephalitis a commonly reported complication of KFD in children [20]. While it is impossible to decipher the exact cause of our patient’s neurologic symptoms, KFD could have well been a contributing factor, and her simultaneous affliction with KFD could not be entirely coincidental.

## Conclusions

KFD is a rare and poorly described disease with clinical features closely mimicking hematologic malignancies or autoimmune disorders. However, unlike malignancies or autoimmune disorders, it is a benign and self-limited syndrome best managed by observation and supportive care. Because of its non-specific symptoms and variable serologic findings, clinicians often misdiagnose KFD as a viral infection, autoimmune disorder, or hematologic malignancy. This can lead to unnecessary and often invasive diagnostic tests and the initiation of unwarranted therapies. Consequently, it is imperative for clinicians to be familiar with KFD and suspect it in young patients, especially those with a history of systemic autoimmune diseases, who present with subacute onset of tender lymphadenopathy, low-grade fevers, and leukopenia in the absence of other underlying etiologies. To the best of our knowledge, this is the first reported case of a patient with KFD associated with RA, Hashimoto’s hypothyroidism, and DM-1.
